# Iron-Based Superconductors for High-Field Applications: Realization of High Engineering Critical Current Density

**DOI:** 10.3390/ma17215306

**Published:** 2024-10-31

**Authors:** Peng Yang, He Huang, Meng Han, Cong Liu, Chao Yao, Yanwei Ma, Dongliang Wang

**Affiliations:** 1 Key Laboratory of Applied Superconductivity, Institute of Electrical Engineering, Chinese Academy of Sciences, Beijing 100190, China; 2 University of Chinese Academy of Sciences, Beijing 100049, China; 3 Institute of Electrical Engineering and Advanced Electromagnetic Drive Technology, Qilu Zhongke, Jinan 250013, China

**Keywords:** iron-based superconductors, engineering critical current density, superconducting tapes

## Abstract

Iron-based superconductors have strong potential for magnet applications through their very high upper critical field, low anisotropy and manufacturability through the powder-in-tube (PIT) route. The engineering critical current density (*J*_e_) is a key parameter for measuring the maximum current density that superconducting materials can withstand in practical applications. It serves as a bridge between theoretical research and practical applications of superconductors and has great significance in promoting the development and application of superconducting technology. In this study, Ag sheathed Ba_0.6_K_0.4_Fe_2_As_2_ (Ba-122) iron-based superconducting tapes were prepared by using the process of drawing, flat rolling and heat treatment by hot pressing (HP). For the first time, the filling factor of the tapes increased to about 40%, leading to a reduction in the volume fraction of Ag, consequently lowering the overall cost. The optimal parameters for achieving high transport *J*_e_ were obtained by comparing the effects of different HP pressures on the properties and micro-morphology of the tapes. The prepared mono-filament tapes are capable of carrying the transport *J*_e_ of 4.1 × 10^4^ A/cm^2^ (*I*_c_ = 350 A) at 4.2 K, 10 T, marking the highest *J*_e_ reported for Ba-122 wires and tapes to date. Our results show that high transport *J*_e_ can be obtained in Ba-122 superconducting tapes, and iron-based superconductors have a promising future in practical applications.

## 1. Introduction

For current high-field applications such as high-field magnets, nuclear magnetic resonance, nuclear fusion [1,2,3], superconducting long wires and tapes with high transport currents are required. As a new type of superconducting material, iron-based superconductors have had important potential applications in the high field since their discovery [4,5,6,7] due to their high upper critical field [8], high transition temperature [9,10] and small anisotropy [11]. In addition, iron-based superconductors can be fabricated using the low-cost PIT method, making it possible to produce large-scale and practical superconducting long wires and tapes [12,13,14]. In recent years, the critical current density (*J*_c_) of Ba_0.6_K_0.4_Fe_2_As_2_ (Ba-122) superconducting wires and tapes made by the powder-in-tube (PIT) method has continuously improved [15,16]. The engineering critical current density (*J*_e_) is more important for the practical applications after taking into account the geometry of the superconducting material, cooling conditions, magnetic field distribution and other factors. Therefore, it becomes increasingly important to increase the transport *J*_e_ of Ba-122 tapes as improvements in the critical current density continue. Table 1 lists the transport *J*_c_ and *J*_e_ for various typical samples.

There are two main ways to increase transport *J*_e_: one is to increase the transport *J*_c_ using hot pressing (HP), cold pressing (CP) and hot isostatic pressing (HIP), and the other is to increase the filling factor [25,26,27]. As can be seen from Table 1, Ag sheath exhibits superior compatibility with iron-based superconductors [28,29]. Mono-filamentary exhibits a higher transport *J*_c_ compared to multi-filamentary, while tapes consistently demonstrate a higher critical current density than wires. Currently, the highest transport *J*_c_ of Ba-122 tapes has been achieved at 1.5 × 10^5^ A/cm^2^ using the HP method [18]. Despite the significant advancements made by both multi-filamentary and composite sheath materials, such as Cu/Ag [30] and SS/Ag [19,31], in enhancing the transport *J*_c_, the relatively lower filling factor is still a limiting factor for transport *J*_e_. Mono-filamentary samples, characterized by a higher filling factor, offer the potential for achieving higher transport *J*_e_. At present, the filling factor for Ag-sheathed wires and tapes is about 20–25%, and for composite-sheathed wires and multi-filamentary wires the filling factor is even lower, so improving *J*_e_ by employing metal tubes with a thinner sheath and finer diameter to produce tapes is an indispensable step. However, it is worth noting that only focusing on increasing the filling factor could potentially result in a reduction in *J*_c_ in the tapes. This is because a high initial density can adversely impact the flowability of the superconducting powders during the cold working process, which in turn may compromise the overall performance of the sample [32]. In addition, variations in the filling factor can cause changes in the processing parameters, microstructure and grain size, thus affecting the performance of the tapes. The process of HP can be used to mitigate this phenomenon but requires further improvement. Therefore, optimizing the HP process to balance the negative impact of the increase in filling factor is a key strategy for the preparation of Ba-122 tapes with high transport *J*_e_.

In this paper, we prepared Ag sheathed Ba-122 tapes with higher transport *J*_e_ by the HP method. The use of a thinner sheath and higher initial tube density effectively increased the filling factor of the tapes to about 40%. By optimizing the preparation process, the transport *J*_e_ of the Ba-122 tapes reaches 4.1 × 10^4^ A/cm^2^ at 4.2 K and 10 T, which is the highest transport *J*_e_ for Ba-122 tapes so far. The results show that it is possible to achieve a high filling factor and high *J*_e_ in Ba-122 iron-based superconducting tapes, which is of great significance for practical applications of iron-based superconducting tapes.

## 2. Experimental Methods

**Figure 1:**
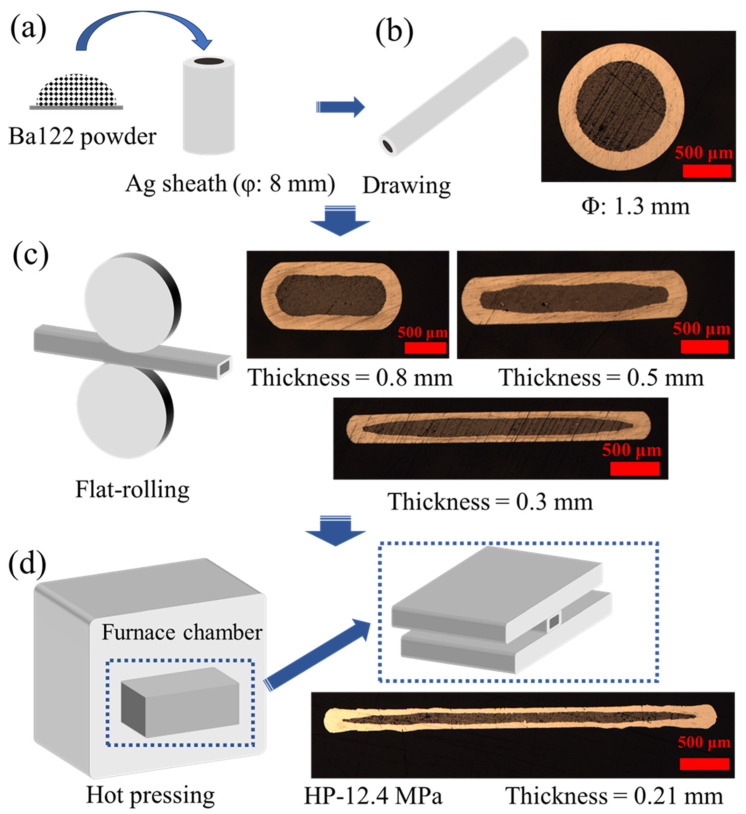
Schematic diagram of the fabrication process of Ag sheathed Ba-122 tapes and cross-sectional optical images of the sample for different processes. (**a**) After grinding, the precursor was loaded into the Ag tube. (**b**) The composite Ag tube was processed into a wire with an outer diameter of 1.3 mm by drawing. (**c**) The Ag wire was rolled into a tape with a thickness of 0.3 mm. (**d**) The rolled tapes were sintered using the HP method.

A Ba-122 superconductor was prepared by the solid-state reaction method. In order to avoid insufficient elemental reactions, a Ba-122 superconductor was prepared by a two-step method. Firstly, the intermediate BaAs and KAs were prepared in advance by mixing Ba, As and K elements. Then, the intermediates were mixed with Fe and As powders using ball milling and sintered at 900 °C for 35 h in a quartz tube furnace. The sintered material is a Ba-122 precursor. It was loaded into a silver tube (outer diameter: 8 mm, inner diameter: 6 mm) after grinding the precursor. The filling factor in the tapes was improved by compacting the powder into tubes. The composite Ag tube was processed into a wire with an outer diameter of 1.3 mm by drawing, and then the wire was rolled into a tape with a thickness of 0.3 mm by flat rolling; the whole process is shown in Figure 1. The optical image of the tape cross-section during each rolling process is also shown in Figure 1. The rolled tape was cut into a series of short tapes and sintered at 880 °C for 1.5 h in a muffle furnace, denoted by Tape-0 in this paper. The HP process was adopted for the other tapes with different pressures at 880 °C for 1.5 h. Different pressures of 7.0 MPa (200 kg), 10.3 MPa (300 kg), 12.4 MPa (400 kg), 15.4 MPa (500 kg) and 18.3 MPa (600 kg) were applied to the tapes for 1 h during the sintering process, denoted by HP-7.0 MPa, HP-10.3 MPa, HP-12.4 MPa, HP-15.4 MPa and HP-18.3 MPa in this paper, respectively. The high-performance precursor was prepared by using a high-performance glove box (manufactured by Vigor, China), which can monitor lower water and oxygen content. On the basis of optimizing pressure, we further used the high-performance precursor to improve the transport *J*_e_, denoted by HP_H_-12.4 MPa.

The field-dependent transport current was measured by using the standard four-lead method at the Institute of Plasma Physics, CAS, in Hefei, with the criterion of 1 μV/cm. The short sample of Ba122 tapes was inserted into conductive resin and then polished for optical microscopic observation and Vickers hardness measurements. The Vickers hardness values of the superconducting cores were measured on the transverse cross-sections with a 0.025 kg load and 10 s duration using the Vickers hardness tester (402MVD, Wolpert Wilson Instruments, Boston, MA, USA). The phase structures of the precursor powder and the tape were tested by X-ray diffraction (XRD, Bruker D8 Advance, Karlsruhe, Germany). The grain morphology of the samples was observed using a scanning electron microscope (SEM, SU8600, Tokyo, Japan). The magnetic property of the tapes was evaluated by a SQUID-VSM on a magnetic property measurement system (MPMS, Quantum Design, San Diego, CA, USA). The crystal orientation and grain size were analyzed by an electron backscatter diffraction (EBSD) plugin equipped on a scanning electron microscope (DIGIVIEW 5, EDAX, Pleasanton, CA, USA).

## 3. Results and Discussion

The parameters of the tapes are listed in Table 2 to compare the effect of pressure on the tapes. The filling factor is the ratio of the superconducting area to the total area on the transverse cross-section of tapes. The critical current density (*J*_c_) is determined by *J*_c_ = *I*_c_/*S*_SC_, and the engineering critical current density (*J*_e_) is given by *J*_e_ = *I*_c_/*S*_Total_, where *I*_c_ is the transport current measured by the four-lead method, and *S*_SC_ and *S*_Total_ represent the area of the superconducting core and the total area of the tapes, respectively. As shown in Table 2, with the increase in pressure, the thickness of the tapes initially decreases significantly, and then the deformation gradually decreases. The width of the sample gradually increases with increasing pressure. The fill factor of the tapes is around 40%, with a maximum value of 41.0%. To quantify the amount of deformation caused by HP, the reduction ratio *r* can be defined as *r* = (*D*_0_ − *d*)/*D*_0_, *D*_0_ is the initial thickness, and *d* is the final thickness. The *r* values of the tapes can be obtained as 0.13, 0.19, 0.32, 0.29, 0.35 and 0.39, respectively.

**Figure 2:**
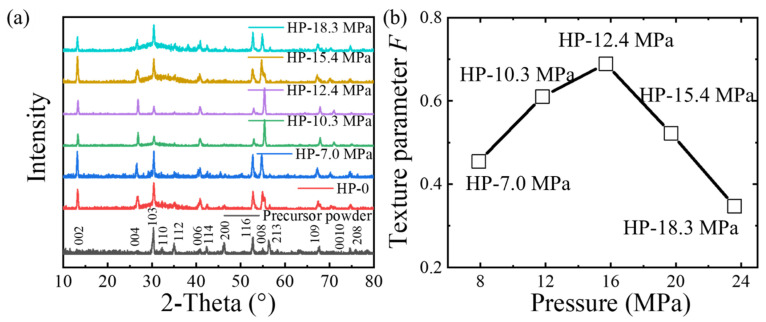
(**a**) XRD patterns of precursor powder and the cores of the Ba-122 tapes. (**b**) The Lotgering orientation factor *F* as a function of the pressure.

The XRD patterns of the Ba-122 tapes are shown in Figure 2a, and the non-oriented precursor powders are also included for comparison. From the results, it can be seen that there are no obvious impurity peaks except for the Ba-122 phase, indicating that the sample has good phase purity. Comparing the XRD results of the tapes and precursor, it can be seen that the 00*l* peak has been significantly strengthened, indicating that the tapes have a good *c*-axis texture. Some of the high-angle peak shifts may be due to increased pressure. The Lotgering method [33] can be used to quantitatively calculate the texture factor $F=(\rho -\rho_{0})/(1-\rho_{0})$, where ρ=∑I(00l)/∑I(hkl), $\rho_{0}=\sum I_{0}(00l)/\sum I_{0}(hkl)$, where I and $I_{0}$ represent the peak intensities of the Ba-122 tapes and randomly oriented powder, respectively. The variation in the *F* value of the tapes with pressure after calculation is shown in Figure 2b. From the results, it can be seen that as the pressure increases, the texture of the sample first increases and then decreases. At 12.4 MPa, the texture factor reaches its maximum value of 0.68. This value is lower than the reported *F* = 0.87 of HP Ba-122 tapes [18], and the decrease in *F* may be the reason for the decrease in transport *J*_c_. The decrease in the *F* with increasing pressure may be related to the inhomogeneity of the stress distribution on the sample cross-section. In addition, the density of superconducting cores is also an important parameter related to the performance of the tapes. Vickers hardness can effectively reflect the density of superconducting cores in tapes. The hardness of the samples was measured using a Vickers hardness tester. The average values for tapes HP-7.0 MPa, HP-10.3 MPa, HP-12.4 MPa, HP-15.4 MPa and HP-18.3 MPa are 132.2, 138.3, 153.0, 150.1 and 149.2, respectively. From the above results, we can find that the high degree of texturing and density of superconducting cores determines the high performance of Ba-122 tapes.

**Figure 3:**
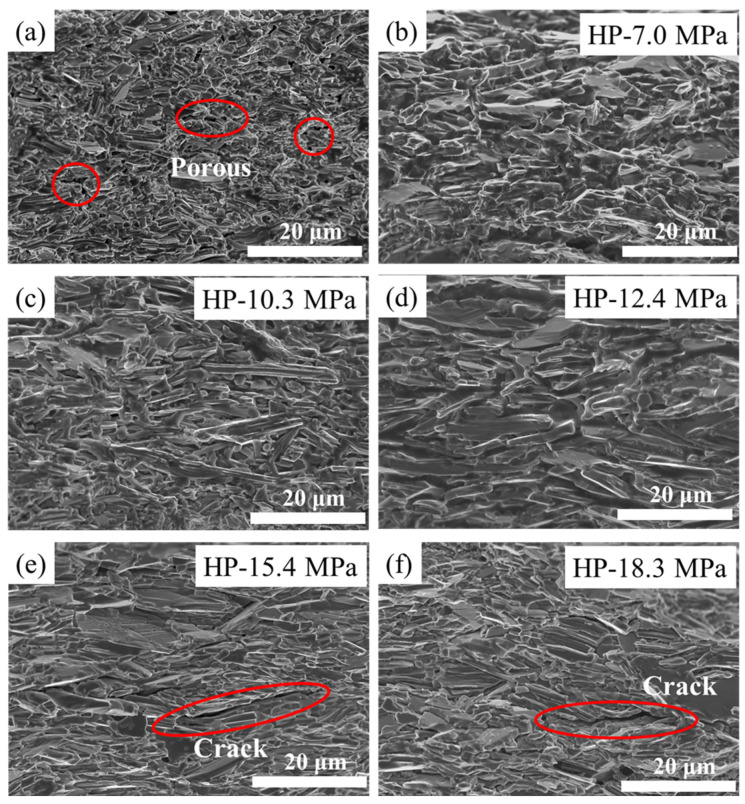
SEM images of the superconducting cores for the tapes under different pressures (**a**) The tape without HP method exhibits pores within its center, as highlighted by the red circle in the figure. (**b**) HP-7.0 MPa, (**c**) HP-10.3 MPa, (**d**) HP-12.4 MPa, (**e**) HP-15.4 MPa and (**f**) HP-18.3 MPa. The red circles indicate cracks in the tapes.

The influence of different pressures on the microstructure of the superconducting tapes was obtained by conducting an SEM analysis on the Ba-122 superconducting core (as shown in Figure 3). The results show that the tape without HP has smaller grains and some areas have pores, which may be the reason for the lower performance. The phenomenon of pores appearing in the flat rolled tapes has also been reported in [34]. From Figure 3b–f, it can be seen that hot pressing can effectively reduce the porosity in superconducting cores and promote the growth of superconducting grains. The size of superconducting grains in the tapes first increases significantly (Figure 3b–d) and then gradually becomes similar in size (Figure 3d–f) as the pressure increases. In addition, it can be found that the arrangement of grains in the tapes has also been significantly improved with the increase in pressure. However, the microcracks appear in the local area of the superconducting core along the width direction when the pressure is too high, which may be due to the excessive pressure, resulting in transverse slip of the superconducting grains. From the results, it can be seen that HP-12.4 MPa has a good microstructure, which may be one of the reasons for its superior performance.

**Figure 4:**
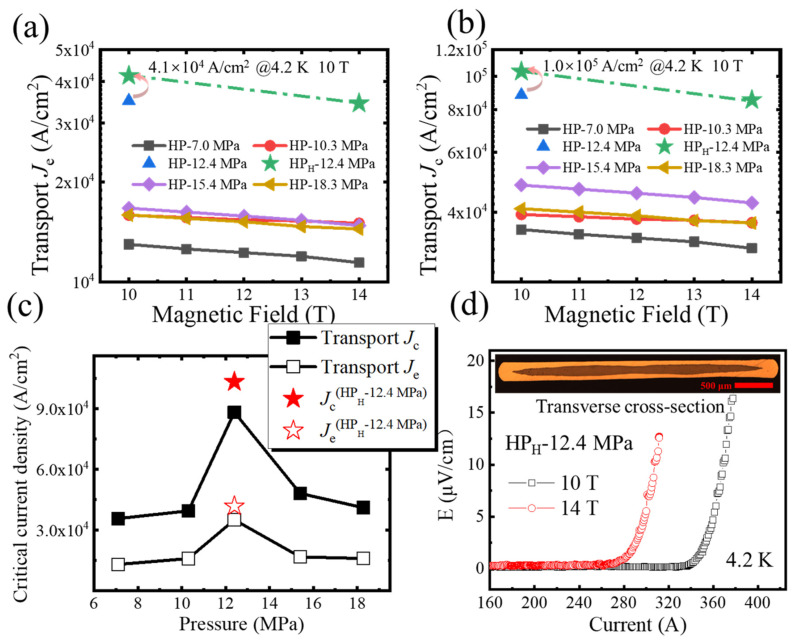
(**a**,**b**) The magnetic field dependence of transport *J*_e_ and transport *J*_c_ for tapes under different HP pressures, respectively. (**c**) The results of transport *J*_e_ and transport *J*_c_ for Ba-122 tapes with different HP pressures at 4.2 K, 10 T, respectively. (**d**) *E-J* curves under various magnetic fields at 4.2 K of Ba-122 tape (HP_H_-12.4 MPa).

Figure 4a depicts the field dependence of transport *J*_e_ at different pressures. The results indicate that at 4.2 K and 10 T, the transport *J*_e_ of the HP-12.4 MPa sample is relatively high compared to tapes prepared using other HP pressures, reaching 3.5 × 10^4^ A/cm^2^. Through further repeated experiments using the high-performance precursor, we have further enhanced the *J*_e_ of Ag sheathed Ba-122 tapes to 4.1 × 10^4^ A/cm^2^ at 4.2 K, 10 T (as shown in Figure 4a), which is currently the highest transport *J*_e_. This value exceeds 37% of the reported value of the flat-rolled Ba-122 tapes prepared by the HP method [18]. At 4.2 K and 14 T, the transport *J*_e_ can reach 3.4 × 10^4^ A/cm^2^. Figure 4b shows the magnetic field dependence of transport *J*_c_, where the *J*_c_ value of the HP-12.4 MPa tape reached 8.8 × 10^4^ A/cm^2^ at 4.2 K, 10 T. The transport *J*_c_ of the tape prepared using the high-performance precursor reaches 1.0 × 10^5^ A/cm^2^ at 4.2 K and 10 T. Figure 4c shows the curve of the transport *J*_e_ and *J*_c_ of the tapes as a function of pressure at 4.2 K and 10 T. It can be seen from the figure that the transport *J*_e_ and *J*_c_ of the Ba-122 tapes first increases and then decreases with pressure, reaching its maximum value at 12.4 MPa, which is different from the already reported 15 MPa [18]. Due to the use of a small drawing diameter and high filling factor in the preparation process, *J*_c_ is relatively low and *J*_e_ is high in the present work, as compared to [18]. In addition, due to the thin sheath, the tape is damaged after the transport current test is completed due to the high *I*_c_. The electric-field–current density (*E–J*) curves of the high-performance tape (HP_H_—12.4 MPa) at 4.2 K under different magnetic fields were obtained through transport *I*_c_ measurement, as shown in Figure 4d. The *I*_c_ of the tape reaches 350 A at 4.2 K and 10 T. The inset is a transverse cross-sectional optical microscope image of the tape. The tape with high transport *J*_e_ has a thickness of 0.22 mm, a width of 3.68 mm, a total area *S*_Total_ = 0.84 mm^2^ and a filling factor of about 40%. The *n*-value of the tape at 10 T is 28.2, indicating a more nonuniform distribution of transport current. This may be due to the large proportion of superconducting cores, which affects the uneven microstructure and grain connectivity defects inside the superconducting cores [35].

**Figure 5:**
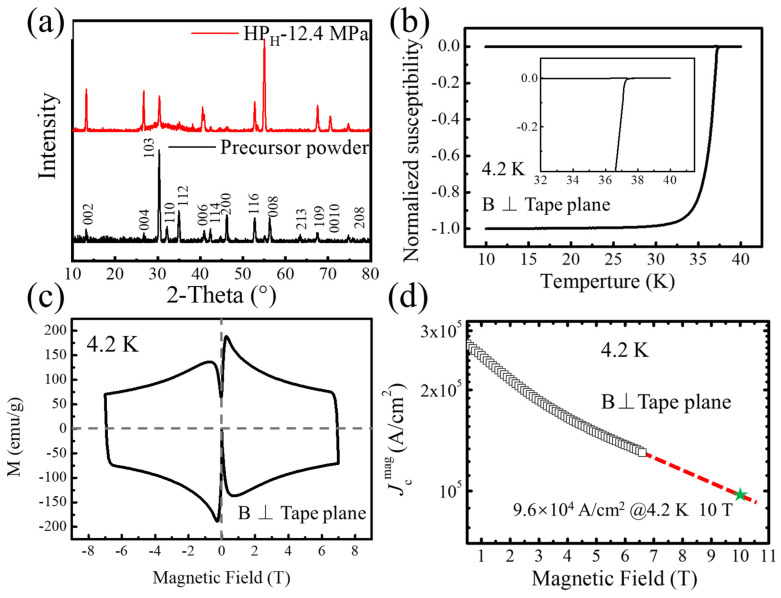
(**a**) XRD patterns for the superconducting cores of tapes (HP_H_-12.4 MPa) and randomly orientated precursor powder. (**b**) The normalized susceptibility as a function of temperatures of the Ba-122 tape (HP_H_-12.4 MPa). (**c**) Magnetization hysteresis loops of the Ba-122 tape (HP_H_-12.4 MPa). (**d**) $J_{c}^{mag}$–*B* curves (HP_H_-12.4 MPa) calculated via the Bean model. The green star indicates the $J_{c}^{mag}$ of the tape at 4.2 K and 10 T as deduced from the calculations.

In order to link the high transport *J*_e_ in the tape to their microstructures, detailed characterizations were carried out on the sample HP_H_-12.4 MPa. Figure 5a shows the XRD results of the tape and Ba-122 precursor, and the calculated *F* value is 0.64, which is close to the results of HP-12.4 MPa. Figure 5b shows the *M-T* (magnetization as a function of temperature) curves of the sample. The transition temperature is 37.8 K, and the sharp transition indicates the high-quality Ba-122 phase in the tapes. Figure 5c shows the *M*-*H* (magnetization as a function of magnetic field) curve of the field perpendicular to the surface of the tape at 4.2 K. A large hysteresis loop indicates the presence of a significant global current throughout the entire tape. The magnetic *J*_c_ of the tapes was calculated via the Bean model:

Jcmag=20∆Ma(1−a3b)

where $J_{c}^{mag}$ is the magnetic critical current density, ∆*M* is the difference between the magnetization for increasing and decreasing fields, and *a* and *b* are the width and length of the sample with *a* < *b*. As shown in Figure 5d, at 4.2 K and 6 T, the $J_{c}^{mag}$ of the tape can reach 1.3 × 10^5^ A/cm^2^, and the $J_{c}^{mag}$ of the tape is about 9.6 × 10^4^ A/cm^2^ at 4.2 K and 10 T, which is very close to the value of transport *J*_c_.

**Figure 6:**
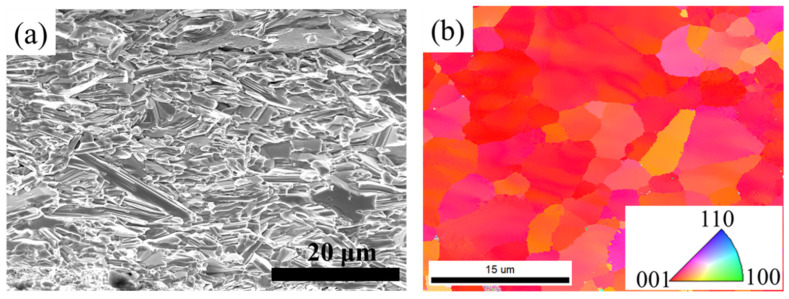
(**a**) SEM and (**b**) IPF map of the Ba-122 tape (HPH-12.4 MPa).

**Figure 7:**
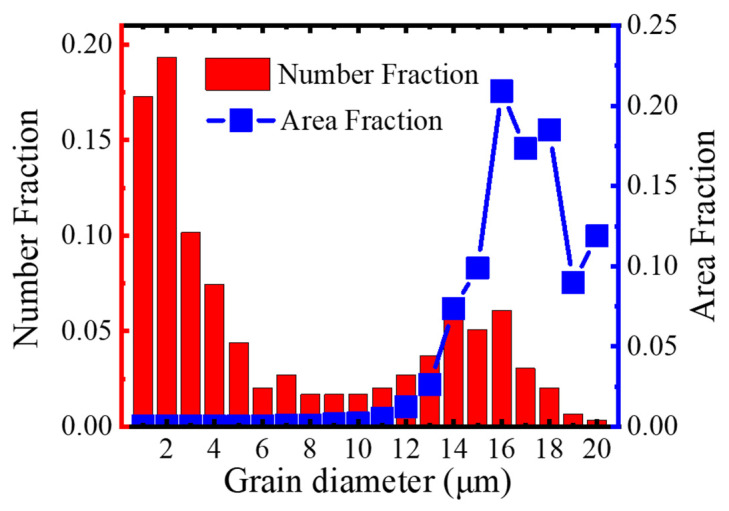
Number fraction and area fraction of grains as a function of grain diameter for Ba-122 tape (HPH-12.4 MPa).

Further observation and characterization of the microstructure of the tape were carried out. Figure 6a shows the SEM image of the sample cross-section, which displays a high density, similar to the results shown in the previous HP-12.4 MPa. Although the XRD and SEM results strongly demonstrate the *c*-axis texture of Ba-122 grains in the tape, quantitative and visual characterization using EBSD technology can further provide valuable information. Figure 6b is an inverse pole figure (IPF) of the tape. The color code is shown in the illustration. The results show that the main color of the tape is red, indicating that the tape has a high *c*-axis texture. Figure 7 presents the statistics of the number fraction and area fraction of grains for the Ba-122 tape. The grain diameter ranges from 0.1 to 20 µm, which is larger than that reported in [18]. The number of grains with a diameter around 2 µm is higher, and the area of large-sized grains accounts for a larger proportion of the total area. The grains have a very significant *c*-axis texture due to the induction of texture by HP, as reported in [34].

In summary, we first obtained the optimal HP parameters by comparing the effect of different pressures on the tapes. The tape prepared under optimal pressure had a higher density, better texture and larger grain size by microscopic characterization. Studies found that the magnetic field dependence of the *J*_c_ of an untextured wire can be significantly reduced by reducing the grain size [36]. However, in textured tapes, the tapes with larger grain size have a higher transport *J*_c_ [34]. We obtained the highest transport *J*_e_ through further experiments. However, due to the thin material of the sheath, it is easily damaged during the measurement of transport current. In the future, further research and the use of sheath materials with higher strengths could be carried out to further increase the engineering critical current density of Ba122 tapes.

## 4. Conclusions

In this article, we have fabricated Ag sheathed Ba-122 tapes through the PIT method and HP process. The performance of Ba-122 superconducting tapes prepared under different HP pressures was systematically studied. The use of a thinner sheath and higher initial tube density effectively increased the filling factor of the final tape to about 40%. Reducing the proportion of Ag is also effective in reducing costs. By optimizing the pressure, the transport *J*_e_ values reached 3.5 × 10^4^ A/cm^2^ at 4.2 K and 10 T. Furthermore, the transport *J*_e_ reaches 4.1 × 10^4^ A/cm^2^ at 4.2 K and 10 T by using the high-performance Ba-122 precursor for the preparation of Ag tapes. The high-performance Ba-122 tape has a high degree of *c*-axis texture and superconducting core densities. This is currently the highest reported transport *J*_e_ for the Ba-122 tapes. These findings convincingly illustrate the outstanding potential of iron-based superconductors. We believe that low-cost preparation of iron-based superconducting tapes with high current-carrying capacity will further promote the practical applications of iron-based superconductivity.

## Figures and Tables

**Table 1:** The parameters for the various typical samples.

	Architecture	Sheath	Thermal Treatment	Transport *J*_c_ (A/cm^2^) 4.2 K, 10 T	Transport *J*_e_ (A/cm^2^) 4.2 K, 10 T
Mono-filamentary	(Sr, K)Fe_2_As_2_ Tape [17]	Cu	HP	3.1 × 10^4^	1.0 × 10^4^
	(Ba, K)Fe_2_As_2_ Tape [18]	Ag	HP	1.5 × 10^5^	3.0 × 10^4^
	(Ba, K)Fe_2_As_2_ Tape [19]	SS/AgSn	CP	1.4 × 10^5^	4.6 × 10^3^
	(Ba, K)Fe_2_As_2_ Wire [20]	Cu/Ag	HIP	3.0 × 10^4^	7.2 × 10^3^
	(Ba, Na)Fe_2_As_2_ Wire [21]	Cu/Ag	HIP	7.1 × 10^4^	\
Multi-filamentary	(Sr, K)Fe_2_As_2_ Tape [22]	Ag	HP	6.1 × 10^4^	\
	(Ba, K)Fe_2_As_2_ Tape [23]	Cu/Ag	HIP	4.8 × 10^4^	7.6 × 10^3^
	(Ba, K)Fe_2_As_2_ Wire [24]	Cu/Ag	HIP	2.8 × 10^4^	1.1 × 10^3^

**Table 2:** Summary of sample information.

Sample	Thickness (mm)	Width (mm)	*S*_Total_ (mm^2^)	*S*_SC_/*S*_Total_	Transport *J*_c_ (A/cm^2^) 4.2 K, 10 T	Transport *J*_e_ (A/cm^2^) 4.2 K, 10 T
HP-0	0.31	3.12	0.92	40.0%	6.7 × 10^3^	2.7 × 10^3^
HP-7.0 MPa	0.27	3.48	0.97	37.1%	3.5 × 10^4^	1.3 × 10^4^
HP-10.3 MPa	0.25	3.56	0.81	41.0%	3.9 × 10^4^	1.6 × 10^4^
HP-12.4 MPa	0.21	3.95	0.82	39.8%	8.8 × 10^4^	3.5 × 10^4^
HP-15.4 MPa	0.20	3.96	0.90	37.0%	4.6 × 10^4^	1.7 × 10^4^
HP-18.3 MPa	0.19	4.02	0.82	39.0%	4.1 × 10^4^	1.6 × 10^4^

## Data Availability

The original contributions presented in the study are included in the article, further inquiries can be directed to the corresponding author.
